# Effect of allyl-isothiocyanate on survival and antimicrobial peptide expression following oral bacterial infections in *Drosophila melanogaster*

**DOI:** 10.3389/fimmu.2024.1404086

**Published:** 2024-05-13

**Authors:** Christian Zimmermann, Sonja Dähn, Anika E. Wagner

**Affiliations:** ^1^ Institute of Nutritional Science, Justus Liebig University, Giessen, Germany; ^2^ Centre for Sustainable Food Systems, Justus Liebig University, Giessen, Germany

**Keywords:** allyl-isothiocyanate, anti-microbial peptides, *Drosophila melanogaster*, infection, bioactive plant compound, survival, gene expression

## Abstract

Since infections with antibiotic-resistant bacteria cause increasing problems worldwide, the identification of alternative therapies is of great importance. Plant-derived bioactives, including allyl-isothiocyanate (AITC), have received attention for their antimicrobial properties. The present study therefore investigates the impact of AITC on survival and antimicrobial peptide (AMP) levels in *Drosophila melanogaster* challenged with the fly pathogenic bacteria *Pectobacterium carotovorum* subsp. *carotovorum* and *Leuconostoc pseudomesenteroides*. AITC, a sulfur-containing compound derived from glucosinolates, exhibits antimicrobial properties and has been suggested to modulate AMP expression. By using *D. melanogaster*, we demonstrate that AITC treatment resulted in a concentration-dependent decrease of survival rates among female flies, particularly in the presence of the Gram-negative bacterium *Pectobacterium carotovorum* subsp. *carotovorum*, whereas AITC did not affect survival in male flies. Despite the ability of isothiocyanates to induce AMP expression in cell culture, we did not detect significant changes in AMP mRNA levels in infected flies exposed to AITC. Our findings suggest sex-specific differences in response to AITC treatment and bacterial infections, underlining the complexity of host–pathogen interactions and potential limitations of AITC as a preventive or therapeutic compound at least in *D. melanogaster* models of bacterial infections.

## 1 Introduction

The widespread use of antibiotics in livestock farming and medicine causes increasing problems with antibiotic-resistant pathogenic bacteria (1). In 2019, antibiotic-resistant bacteria have been made responsible for 1.27 million deaths worldwide (WHO) (2). The identification and the development of alternative therapies and prevention strategies is, therefore, of high importance for the global fight against severe bacterial infections. In this context, bioactive plant compounds may be a promising alternative in the treatment of diseases caused by antibiotic-resistant bacteria (3). Plant bioactives are compounds produced within the secondary metabolism of plants, which are used either as a defense mechanism against environmental stressors such as UV radiation, predators, and pathogenic microorganisms (4) or as coloring agents and odorants to attract pollinators (5, 6). Various plant bioactives have been shown to exhibit several health-promoting properties including anti-inflammatory, anti-infectious, anticancer, and antibacterial effects (7–10). Especially the sulfur-containing compounds such as isothiocyanates have been suggested to mediate antibacterial properties (8). In the presence of a neutral milieu, isothiocyanates are generated through hydrolysis from glucosinolates (GLS) by the enzymatic action of myrosinase, a thioglucohydrolase. While the GLS can be found in the vacuoles of cruciferous plant cells, myrosinase is generally present in separate myrosin cells and only gets in contact with GLS following the destruction of the plant cell by, e.g., chewing or cutting (11).

Allyl-isothiocyanate (AITC), the compound which is responsible for the pungent taste of, e.g., wasabi, radish, and mustard, is formed during the myrosinase-catalyzed conversion of the GLS sinigrin following cell disruption. In humans, several biological and health-promoting effects of AITC have been postulated, including anti-inflammatory and antioxidant effects as well as anticancer properties and antimicrobial activities (12–14). In particular, AITC has been able to inhibit the growth of different strains of *Campylobacter jejuni* (8), *Escherichia coli*, and *Listeria monocytogenes in vitro* (15). In cell culture studies, it has also been demonstrated that isothiocyanates are able to increase the expression levels of antimicrobial peptides (AMPs). In particular, it has been shown that the isothiocyanate sulforaphane induces the expression of human β-defensin-2 in intestinal epithelial cells (16). AMPs are known to play a critical role in the innate immune response and are produced by a variety of organisms including mammalian species, insects, and plants (17). They are mainly positively charged proteins and can act by membrane- or non-membrane-targeting mechanisms, thereby destroying bacteria, fungi, or viruses. Since recently, AMPs have been suggested as an alternative treatment option in the therapy of bacterial infections (1). Therefore, we suggest that AITC exhibits its antibacterial effect via increasing the expression levels of AMPs. To test this hypothesis, we applied the fruit fly *Drosophila melanogaster* as a model organism. *D. melanogaster* is especially known for its use in genetic research but has also been well established as a model to investigate and answer physiological questions (18). Due to its short generation time, the relatively inexpensive and easy maintenance, and the fact that 60% of the genes present have orthologs in mammals, the fruit fly is an ideal model for studying evolutionarily conserved genes and signaling pathways (19, 20). Due to the absence of an adaptive immune response in the fruit fly, processes of the innate immunity can be studied individually (21). In *D. melanogaster*, AMPs are produced in the fat body following systemic infections but also to a certain extent in the gut epithelia. The formation of AMPs in *D. melanogaster* is triggered by the activation of the two NFκB pathways, the toll- and the Imd pathway (22). This is highly similar to humans, where the expression of AMPs is also initiated by an activation of toll-like-receptors (23). To activate these pathways in *D. melanogaster*, we orally applied the two different fly-pathogenic bacterial strains *Pectobacterium carotovorum* subsp. *carotovorum* (ECC) and *Leuconostoc pseudomesenteroides* (LP), which have been shown to induce AMP secretion (24–26). ECC is a Gram-negative bacterium; therefore, it is a potential activator of the Imd pathway. LP is a Gram-positive bacterium, potentially activating the toll pathway (27). To test, whether AITC intervenes in these processes we fed flies AITC-supplemented diets, exposed them to the pathogenic bacteria and checked the flies for health-related parameters including survival and AMP expression.

## 2 Materials and methods

### 2.1 Husbandry of w^1118^ *Drosophila melanogaster*

Female and male w^1118^ *D. melanogaster* (Bloomington Drosophila Stock Center, Indiana, USA; #5905) were reared under standard conditions in a climate chamber (Memmert, HPP400, Büchenbach, Germany) at 25°C and 60% humidity with a 12 h/12 h light–dark cycle on Caltech Medium (CT), as described previously (28). For experiments, 3-day-old age-matched flies from synchronized eggs were anesthetized with CO_2_ or on ice and separated according to their sex. Then, 25 flies were transferred into vials containing either the control medium or bacterial suspension. The control medium comprises 10% sucrose (Carl Roth, Karlsruhe, Germany), 10% inactive yeast (Genesee via Kisker, Steinfurt, Germany), 2% agar (Apex via Kisker), and as preservatives 0.3% propionic acid (Carl Roth) and 1.5% tegosept (Apex via Kisker) (Linford et al., 2013). A 1-M stock solution of AITC (Sigma-Aldrich, Taufkirchen, Germany) was prepared in ethanol (abs.) (Merck, Darmstadt, Germany). To investigate the effect of AITC and/or bacteria on food intake, survival, and gene expression, different concentrations of AITC (0.250 mM, 0.125 mM) were added to the control medium. For control medium, the same amount of ethanol (abs.) was added to the food.

### 2.2 Bacterial strains and cultivation

For infection studies, the fly pathogenic bacteria *Pectobacterium carotovorum* subsp. *carotovorum* (ECC) (Leibniz Institute DSMZ – German Collection of Microorganisms and Cell Cultures, Braunschweig, Germany) and *Leuconostoc pseudomesenteroides* (LP) (kind gift from Dr. Kwang-Zin Lee, Fraunhofer Institute for Molecular Biology and Applied Ecology, IME, Giessen, Germany) were grown in LB-Broth (Carl Roth) and MRS-Broth (Carl Roth), respectively. Both bacterial strains used were cultivated in a shaking incubator (B. Braun Biotech International, Melsungen, Germany) under aerobic conditions at 29°C and grown over night. In experiments, both bacterial strains were applied in their stationary growth phase.

### 2.3 Oral infection of w^1118^ *Drosophila melanogaster* with *Pectobacterium carotovorum* subsp. *carotovorum* and *Leuconostoc pseudomesenteroides*

For infection experiments, overnight cultures of ECC and LP were adjusted to an OD of 1 with a 100-mM sterile sucrose solution. Subsequently, 1 ml of the bacteria-sucrose suspension or sterile sucrose solution (control solution) was applied to three layers of cellulose paper lining the bottom of the respective vial. Three-day-old age-matched female and male *D. melanogaster* from synchronized eggs were transferred into these vials (25 flies per vial) and maintained under standard conditions for 18 h (see 2.1).

### 2.4 Gustatory assay

To check for potential effects of AITC on the feeding behavior of *D. melanogaster*, the flies’ food intake was determined by applying the gustatory assay according to Deshpande et al. (29). The flies received either a control or an AITC-supplemented diet for 10 days. On day 10, flies were transferred to control medium with and without AITC (0.125 mM and 0.250 mM) supplemented with 0.2% sulforhodamine B sodium salt (Sigma-Aldrich) and kept under standard conditions (see 2.1) for further 8 h. Then, 20 flies per treatment were transferred in 200 µl PBS (pH 7.4) (Thermo Fisher Scientific, Schwerte, Germany) with 1% Triton X-100 (Sigma-Aldrich) and homogenized in a TissueLyser II (Qiagen, Hilden, Germany) at a frequency of 25 s^−1^ for 6 min. Following, the obtained samples were centrifuged (4,000 g, 5 min) and the fluorescence signal (extinction: 535 nm/emission: 590 nm) of the supernatant was detected in a microplate reader SpectraMax iD3 (Molecular Devices, San Jose, USA). Flies fed unstained food served as controls, and the corresponding values were subtracted from the sample readings. Serial dilutions of sulforhodamine B sodium salt were used to generate a standard curve.

### 2.5 Survival analysis

**Figure 1:**
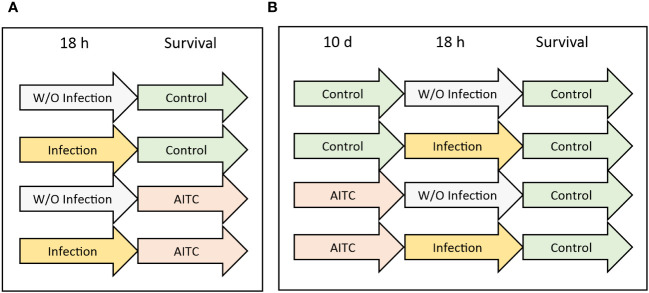
Experimental procedure of survival analysis. **(A)** *D*. *melanogaster* were initially exposed to a 100-mM sucrose solution with or without *L. pseudomesenteroides* or *P. carotovorum* subsp*. carotovorum* for 18 h and were subsequently transferred on either a control or an AITC-supplemented diet (0.125 mM or 0.250 mM) for the rest of their lifetime. **(B)** *D*. *melanogaster* were initially kept on either a control or an AITC-supplemented diet (0.125 mM or 0.250 mM) for 10 days, followed by an exposure to a 100-mM sucrose solution with or without *L. pseudomesenteroides* or *P. carotovorum* subsp*. carotovorum* for 18 h and were subsequently transferred on a control diet for the rest of their lifetime.

In order to investigate an effect of both, bacterial infection and AITC supplementation, on the survival rate of *D. melanogaster*, 3-day-old flies from synchronized eggs were separated according to their sex, sorted into different vials, and exposed to the corresponding treatments as depicted in **Figure 1**. Each experiment was performed two to three times with three vials per sex containing 25 flies each. Every 2–3 days, flies were transferred to new vials with fresh food, whereas dead flies were removed and counted.

### 2.6 RNA-isolation and real-time PCR

**Figure 2:**
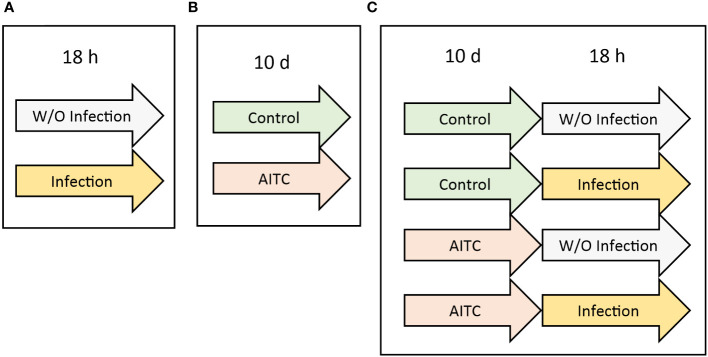
Experimental procedure of generating fly samples for subsequent mRNA analysis. **(A)** *D*. *melanogaster* were initially exposed to a 100-mM sucrose solution with *P. carotovorum* subsp*. carotovorum* or *L. pseudomesenteroides* for 18 h. **(B)** *D*. *melanogaster* were fed either a control diet or an AITC-supplemented diet (0.125 mM or 0.250 mM) for 10 days. **(C)** *D*. *melanogaster* were initially kept on either a control or an AITC-supplemented diet (0.125 mM or 0.250 mM) for 10 days and were subsequently transferred to a 100-mM sucrose solution with or without *L. pseudomesenteroides* or *P. carotovorum* subsp*. carotovorum* for 18 h. .

In order to investigate an effect of both, bacterial infection and AITC supplementation, on mRNA expression 3-day-old flies were exposed to the corresponding treatments as depicted in **Figure 2**. RNA was either isolated by applying TRI Reagent (Ambion, Carlsbad, USA) or the Quick-RNA Tissue/Insect kit (Zymo Research, Freiburg, Germany). In case of RNA isolation with TRI Reagent, five flies were put in a microcentrifuge tube containing 1 ml TRI Reagent and homogenized in a TissueLyser II (Qiagen) at a frequency of 25 s^−1^ for 6 min. After adding 200 µl chloroform, the samples were vortexed and incubated at room temperature for 10 min. Subsequently, the samples were centrifuged at 12,000 g at 4°C for 15 min. Then, the resulting upper, clear phase was transferred into a fresh microcentrifuge tube, mixed with 500 µl ice cold isopropanol, and left on ice for 10 min. Following, the samples were centrifuged at 12,000 g at 4°C for 15 min. The resulting supernatant was discarded, and the remaining pellet was washed twice with 500 µl ice cold 75% ethanol followed by centrifugation at 12,000 g at 4°C for 10 min. After removing the ethanol in the last washing step, the pellets were air-dried at room temperature for 30 min. Following, the dried pellet was resuspended in 50 µl RNase-free-water (Th. Geyer, Renningen, Germany) and dissolved at 55°C for 10 min. Subsequently, RNA samples were subjected to DNase treatment by applying the DNase kit (Sigma-Aldrich) according to the manufacturer’s instructions. In brief, 20 µl of isolated RNA was mixed with 2.5 µl DNase reaction buffer (Sigma-Aldrich) and 2.5 µl DNase I amplification grade (1 U/µl) (Sigma-Aldrich). After 15 min of incubation at room temperature, a 2.5-µl stop solution (Sigma-Aldrich) was added. Then, the samples were heated to 70°C for 10 min. Samples were stored at -80°C until further use.

RNA isolation with the Quick-RNA Tissue/Insect kit (Zymo Research, Freiburg, Germany) was performed according to the manufacturer’s instructions. In brief, 10 flies were homogenized in RNA lysis buffer. After that, the homogenate was transferred into a column tube followed by several washing steps and DNase treatment. Finally, RNA was eluted in DNase/RNase-free water. RNA was stored at −80°C until further use.

The purity of the RNA samples was photometrically detected (260/280 nm) in a UVmini-1240 UV-VIS spectrophotometer (Shimadzu, Duisburg, Germany). Samples with a 260/280-nm ratio lower than 1.7 were excluded from further analysis.

For cDNA synthesis, 1 µg RNA was added to DNase/RNase-free-water, resulting in a total volume of 11.5 µl. Then, 1 µl oligo (dt) primers (20 ng/µl) (Promega, Mannheim, Germany), 4 µl M-MLV RT 5× Buffer (Promega), 0.5 µl RNasin RiboLock (40 U/µl) (Sigma-Aldrich), 2 µl dNTP mix (10 mM) (Promega), and 1 µl M-MLW reverse transcriptase (200 U/µl) (Promega) were added. Afterward, the samples were incubated at 42°C for 60 min and subsequently heated in a thermocycler (T-Gradient ThermoBlock, Biometra, Göttingen, Germany) at 70°C for 10 min. The obtained cDNA was stored at −80°C until further use.

For real-time PCR, cDNA was diluted 1:2 in DNase/RNase-free-water. The master mix contained 10 µl PerfeCTa SYBR Green SuperMix, Low ROX (Quantabio, Beverly, MA, USA), 0.25 µl forward/reverse primer (10 pmol/µl), 7.5 µl DNase/RNase-free-water, and 2 µl cDNA.

Semiquantitative real-time PCR was performed in a 7500 Real-Time PCR system (Applied Biosystems, Heidelberg, Germany). *rPl32* (Ribosomal Protein L32), *RpS20* (Ribosomal Protein S20), and *alphaTub84B* (alpha-Tubulin at 84B) were tested as reference genes. *alphaTub84B* showed treatment-dependent effects, why it was excluded as a reference gene. *rPl32* and *RpS20* were used as reference genes (see for *RpS20* in **Supplementary Table S1**). *Dro* (Drosocin) and *mtk* (Metchnikowin) were measured as target genes (for primer sequences, see **Table 1**).

**Table 1:** Primer sequences for real-time PCR (*Drosophila melanogaster*).

Gene	Forward primer (5′ ➔ 3′)	Reverse primer (3′ ➔ 5′)	Accession no.	Temp.
*dro*	GAGGATCACCTGACTCAAGC	ATGACTTCTCCGCGGTATG	NM_001259395.2	56°C
*mtk*	CCTCATCGTCACCAGGGACC	TTGGACCCGGTCTTGGTTGG	NM_079028.3	55°C
*rPl32*	GGCAAGCTTCAAGATGACCA	GTTCGATCCGTAACCGATGT	NM_170461.3	55°C
*RpS20*	TGTGGTGAGGGTTCCAAGAC	GACGATCTCAGAGGGCGAGT	NM_079697.3	58°C
*alphaTub84B*	TCAGACCTCGAAATCGTAGC	AGCCTGACCAACATGGATAG	NM_057424.4	55°C

dro, Drosocin; mtk, Metchnikowin; rPl32, ribosomal protein L32; RpS20, ribosomal protein S20; alphaTub84B, alpha-tubulin at 84B.

### 2.7 Statistics

Statistical analyses were carried out using GraphPad Prism Software (Version 10.1.2, GraphPad Software, LLC, San Diego, CA, USA). Unless otherwise stated, the experiments were performed in triplicates. Results are presented as mean ± standard deviation (SD) unless otherwise stated. Data were tested for normality of distribution (Shapiro–Wilk) and homogeneity of variances (Brown–Forsythe or Bartlett’s). The means of normally distributed data and homogenous variances were compared by one-way ANOVA followed by a *post hoc* test (Tukey’s) for multiple comparisons. Data with heterogeneous variances were tested with Brown–Forsythe–Welch ANOVA followed by a *post hoc* test (Dunnet’s T3) for multiple comparisons. Not normally distributed data were tested with a non-parametric test (Kruskal–Wallis). Survival analysis was performed by the Kaplan–Meier approach and a log-rank test to test for significant differences. Median survival times were calculated and tested for significant differences by the Mann–Whitney test. Significance was accepted at p < 0.05.

## 3 Results

### 3.1 Effect of AITC on food intake of w^1118^ *Drosophila melanogaster*

**Figure 3:**
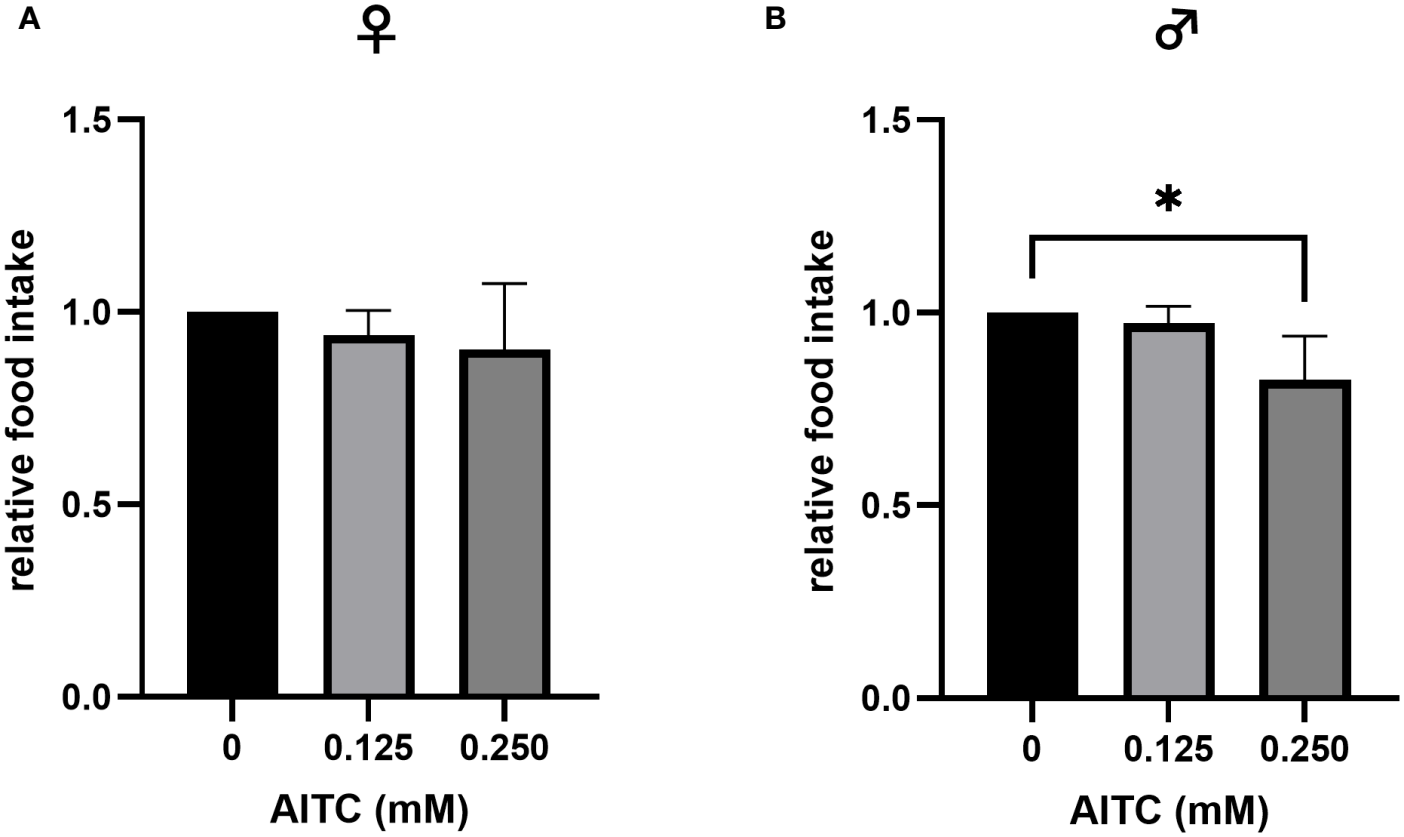
Relative food intake of female **(A)** and male **(B)** *D*. *melanogaster* fed either a control diet or an AITC-supplemented diet (0.125 mM or 0.250 mM), respectively, for 10 days. Bars show the mean ± SD of three independent experiments. Significant differences between groups were tested by applying one-way ANOVA followed by Dunnett’s multiple-comparison test. Significance was accepted at p < 0.05. *p < 0.05.

To test whether AITC enrichment at different concentrations has an effect on food intake in *D. melanogaster*, a gustatory assay was performed. The application of 0.125 mM AITC for 10 days did not affect the flies’ food intake, either in female or in male *D. melanogaster*. In contrast, addition of 0.250 mM AITC significantly reduced the food intake in male flies by approximately 7% but not in female flies (**Figures 3A, B**).

### 3.2 Effect of AITC-supplemented diet and bacterial infection on survival of w^1118^ *Drosophila melanogaster*

**Figure 4:**
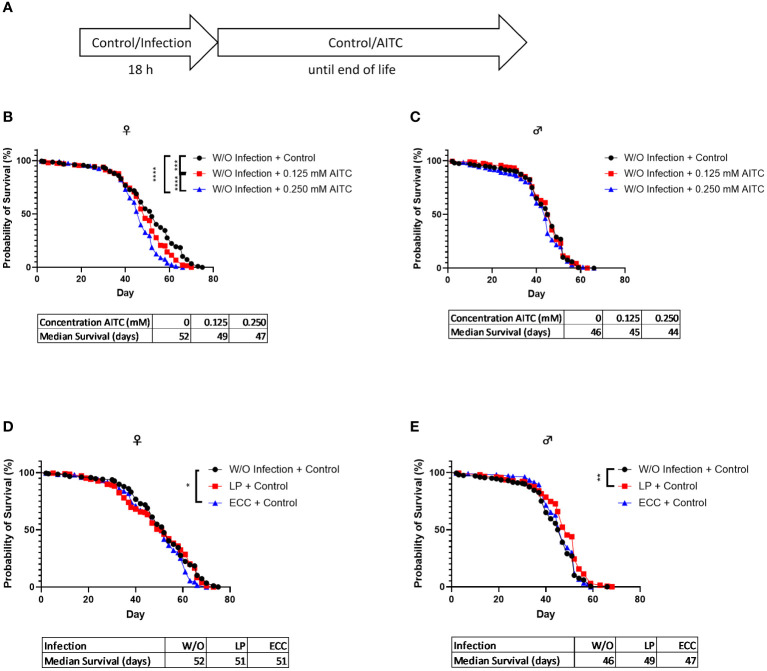
Survival curves and median survival time of female **(B, D)** and male **(C, E)** *D. melanogaster* initially exposed to a control solution (W/O Infection) **(B, C)** or a solution with *L. pseudomesenteroides* (LP) or *P. carotovorum* subsp*. carotovorum* (ECC) **(D, E)** for 18 h and subsequently transferred on either a control or an AITC-supplemented diet (0.125 mM or 0.250 mM) for the rest of their lifetime. The experiment procedure is shown in **(A)**. Results show the three independent experiments. Significant differences between treatments were tested by the Kaplan–Meier approach and a log-rank test. Significance was accepted at p < 0.05. **p < 0.01, ***p < 0.001, ****p < 0.0001.

**Figure 5:**
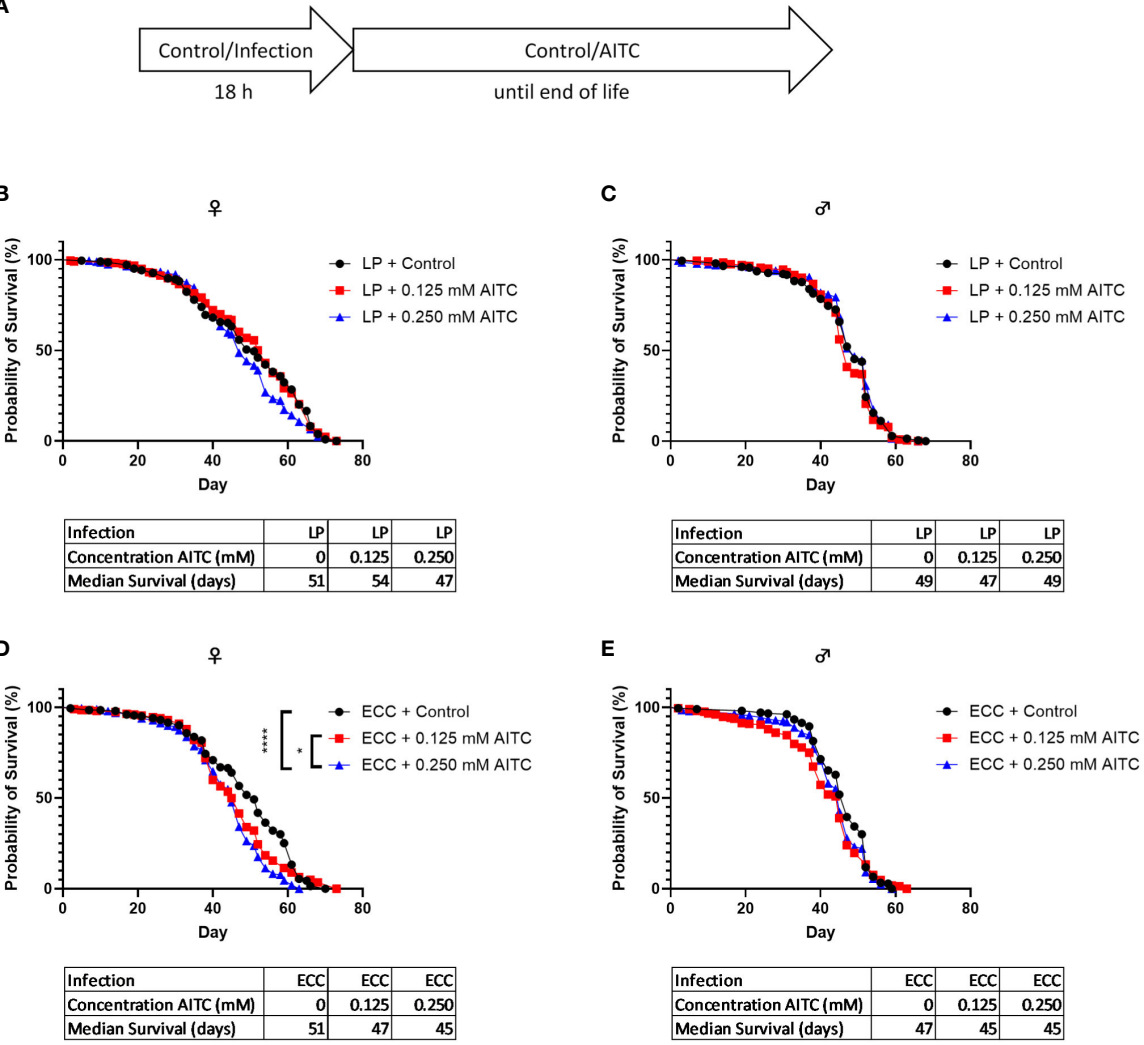
Survival curves and median survival time of female **(B, D)** and male **(C, E)** *D. melanogaster* initially exposed to a solution with *L. pseudomesenteroides* (LP) **(B, C)** or *P. carotovorum* subsp*. carotovorum* (ECC) **(D, E)** for 18 h and subsequently transferred on either a control or an AITC-supplemented diet (0.125 mM or 0.250 mM) for the rest of their lifetime. The experiment procedure is shown in **(A)**. Results show three independent experiments. Significant differences between treatments were tested by the Kaplan–Meier approach and a log-rank test. Significance was accepted at p < 0.05. *p < 0.05, ****p < 0.0001.

A survival assay was performed to investigate potential effects of both bacterial infection and AITC supplementation, on the flies’ survival rates. *D. melanogaster* initially exposed for 18 h to a 100-mM sucrose solution and subsequently transferred on an AITC-supplemented (**Figure 4A**) diet led to a significant concentration-dependent decrease of survival time of female flies. In contrast, no effects of AITC on male flies were observed. The medium survival times did not differ between the groups (**Figures 4B, C**). In female flies, infection with ECC resulted in a decreased survival whereas no significant effect was observed after LP infection. In contrast, LP infection of male flies resulted in an increased survival whereas ECC infection had no effect on the survival of male flies (**Figures 4D, E**). In *D. melanogaster* infected with either ECC or LP and subsequent exposure to AITC (**Figure 5A**), AITC exhibited a significant effect on survival times in ECC-infected female flies only (**Figures 5B–E**). In this case, the administration of 0.125 mM and 0.250 mM AITC significantly reduced the survival time in a concentration-dependent manner (**Figure 5D**).

**Figure 6:**
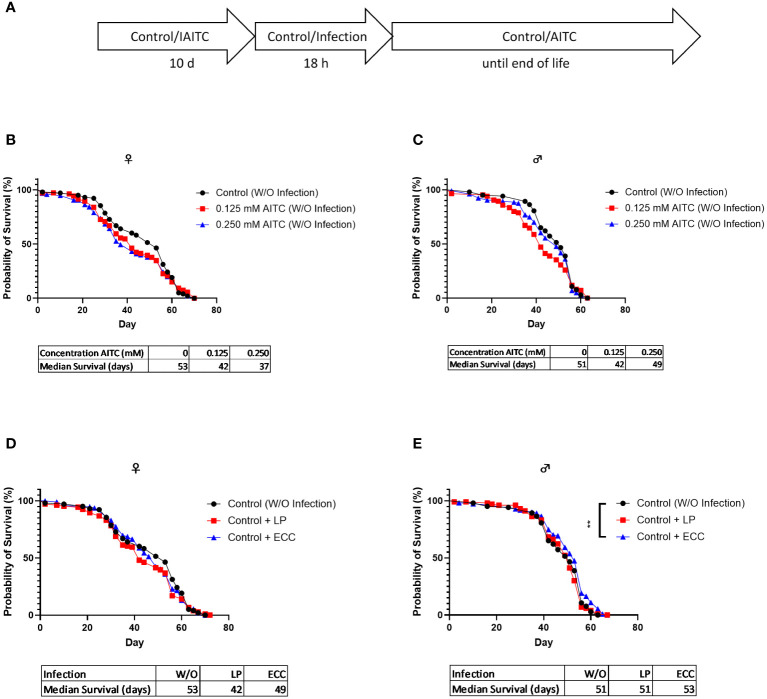
Survival curves and median survival time of female **(B, D)** and male **(C, E)** *D. melanogaster* initially kept on either a control or an AITC-supplemented diet (0.125 mM or 0.250 mM) for 10 days then exposed to a control solution (W/O infection) **(B, C)** or a solution with *L. pseudomesenteroides* (LP) or *P. carotovorum* subsp*. carotovorum* (ECC) **(D, E)** for 18 h and subsequently transferred on a control diet for the rest of their lifetime. The experiment procedure is shown in **(A)**. Results show two independent experiments. Significant differences between treatments were tested by the Kaplan–Meier approach and a log-rank test. Significance was accepted at p < 0.05. **p < 0.01.

**Figure 7:**
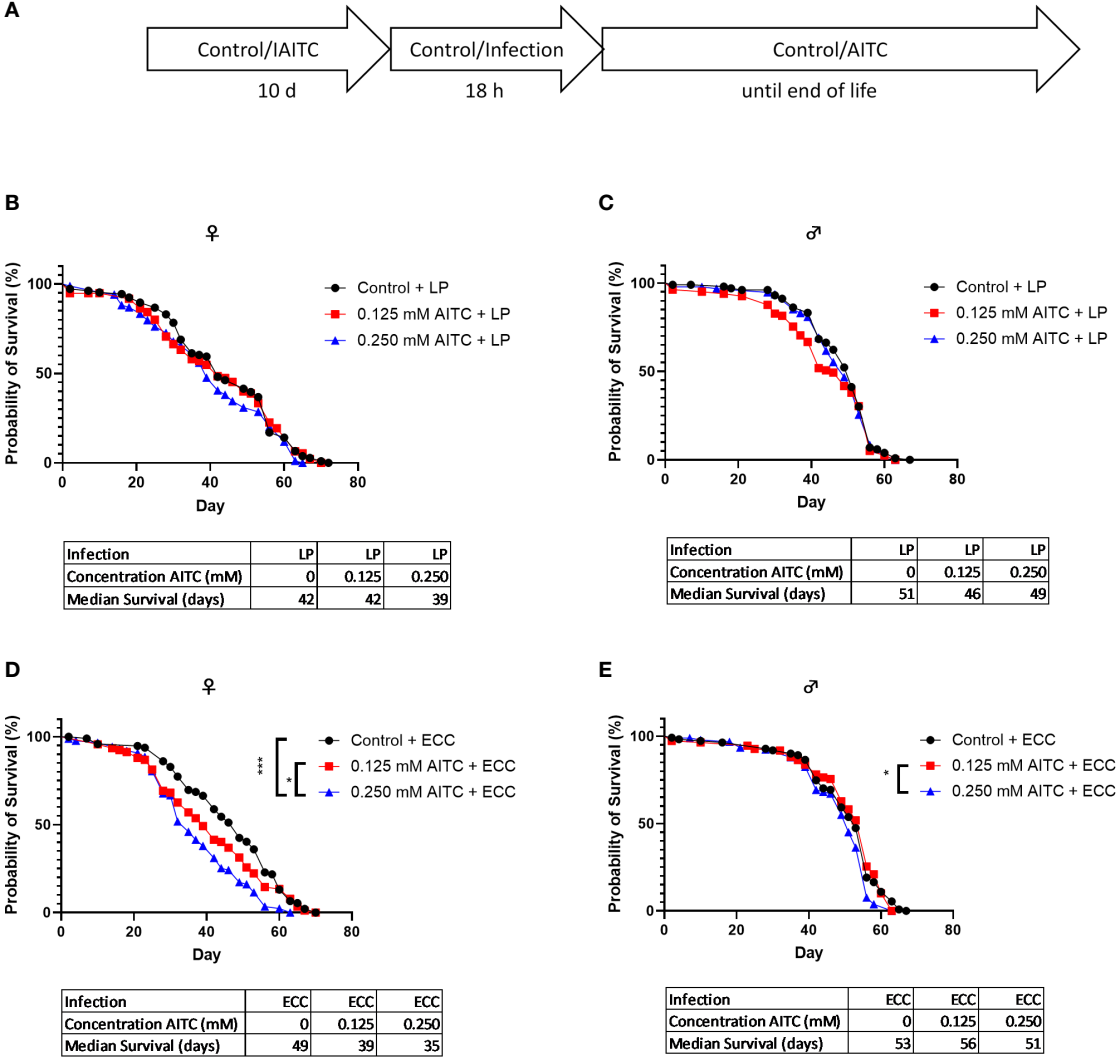
Survival curves and median survival time of female **(B, D)** and male **(C, E)** *D. melanogaster* initially kept either on a control or an AITC-supplemented diet (0.125 mM or 0.250 mM) for 10 days then exposed to a solution with *L. pseudomesenteroides* (LP) **(B, C)** or *P. carotovorum* subsp*. carotovorum* (ECC) **(D, E)** for 18 h and subsequently transferred on a control diet for the rest of their lifetime. The experiment procedure is shown in **(A)**. Results show two independent experiments. Significant differences between treatments were tested by the Kaplan–Meier approach and a log-rank test. Significance was accepted at p < 0.05. *p < 0.05, **p < 0.01, ***p < 0.001.

*D. melanogaster* initially kept on an AITC-supplemented diet for 10 days (**Figure 6A**) did not show any difference in survival rates compared with flies reared on a control diet (**Figures 6B, C**). Both ECC and LP infections of female flies reared on a control diet for 10 days also did not have any effect on the survival rates (**Figure 6D**). In contrast, infection of male flies with ECC resulted in significantly higher survival rates whereas there was no significant difference in the median survival (**Figure 6E**). A pre-feeding with AITC for 10 days (**Figure 7A**) resulted in a concentration-dependent decrease in survival rates of ECC-infected female flies (**Figure 7D**) whereas in ECC-infected male flies, a reduced survival time was observed following exposure of the higher compared with the lower concentration of AITC (**Figure 7E**). AITC pre-feeding did not exhibit any effects on flies infected with LP (**Figures 7B, C**).

### 3.3 Effect of bacterial infections and AITC supplementation on gene expression of antimicrobial peptides in w^1118^ *Drosophila melanogaster*

**Figure 8:**
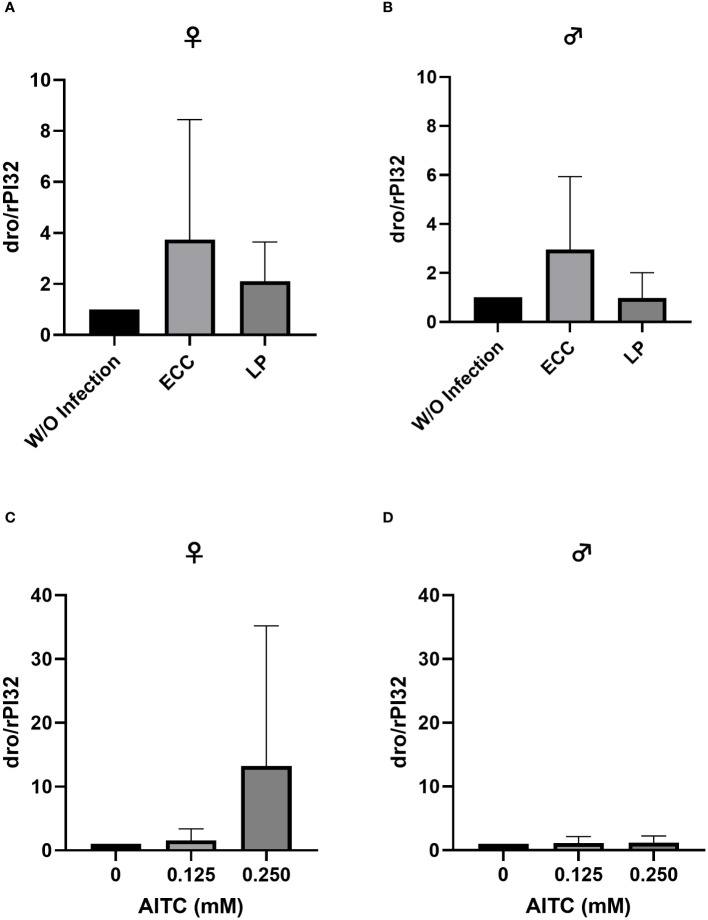
Relative mRNA expression levels of *dro* in female **(A, C)** and male **(B, D)** *D*. *melanogaster* exposed to a 100-mM sucrose solution with *P. carotovorum* subsp*. carotovorum (*ECC*)* or *L. pseudomesenteroides* (LP) for 18 h **(A, B)** and *D*. *melanogaster* fed either a control diet or an AITC-supplemented diet (0.125 mM or 0.250 mM) for 10 days **(C, D)**. mRNA levels were determined in fly samples from three independent experiments with three replicates containing 10 flies each. Bars show the mean ± SD. Significant differences between groups were tested by applying one-way ANOVA followed by Dunnett’s multiple comparison test. Significance was accepted at p < 0.05.

**Figure 9:**
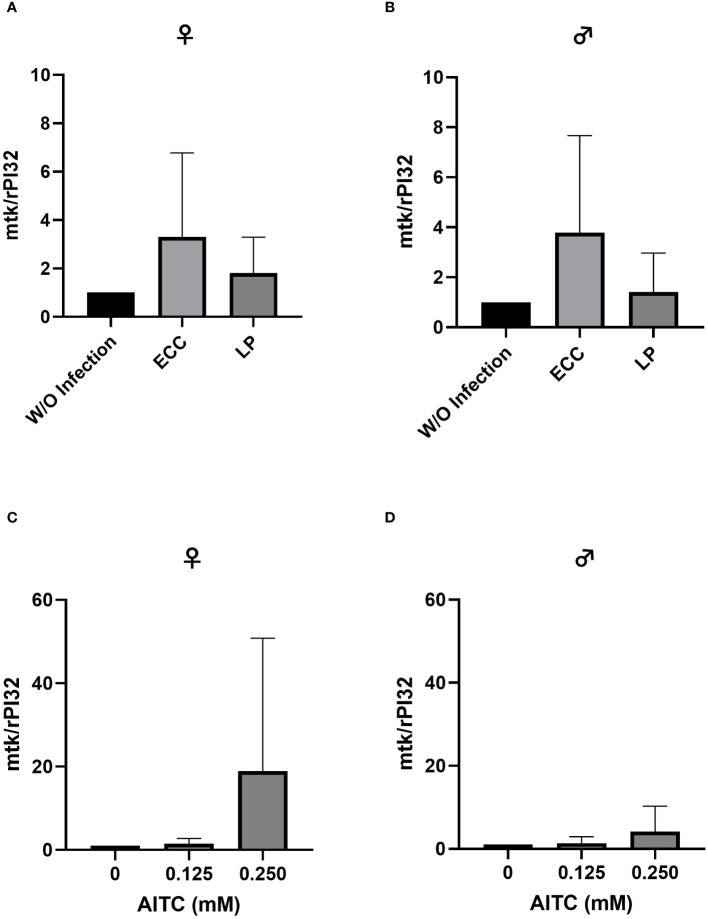
Relative mRNA expression levels of *mtk* in female **(A, C)** and male **(B, D)** *D*. *melanogaster* exposed to a 100-mM sucrose solution with *P. carotovorum* subsp*. carotovorum (*ECC*)* or *L. pseudomesenteroides* (LP) for 18 h **(A, B)** and *D*. *melanogaster* fed either a control diet or an AITC-supplemented diet (0.125 mM or 0.250 mM) for 10 days **(C, D)**. mRNA levels were determined in fly samples from three independent experiments with three replicates containing 10 flies each. Bars show the mean ± SD. Significant differences between groups were tested by applying one-way ANOVA followed by Dunnett’s multiple-comparison test. Significance was accepted at p<0.05.

**Figure 10:**
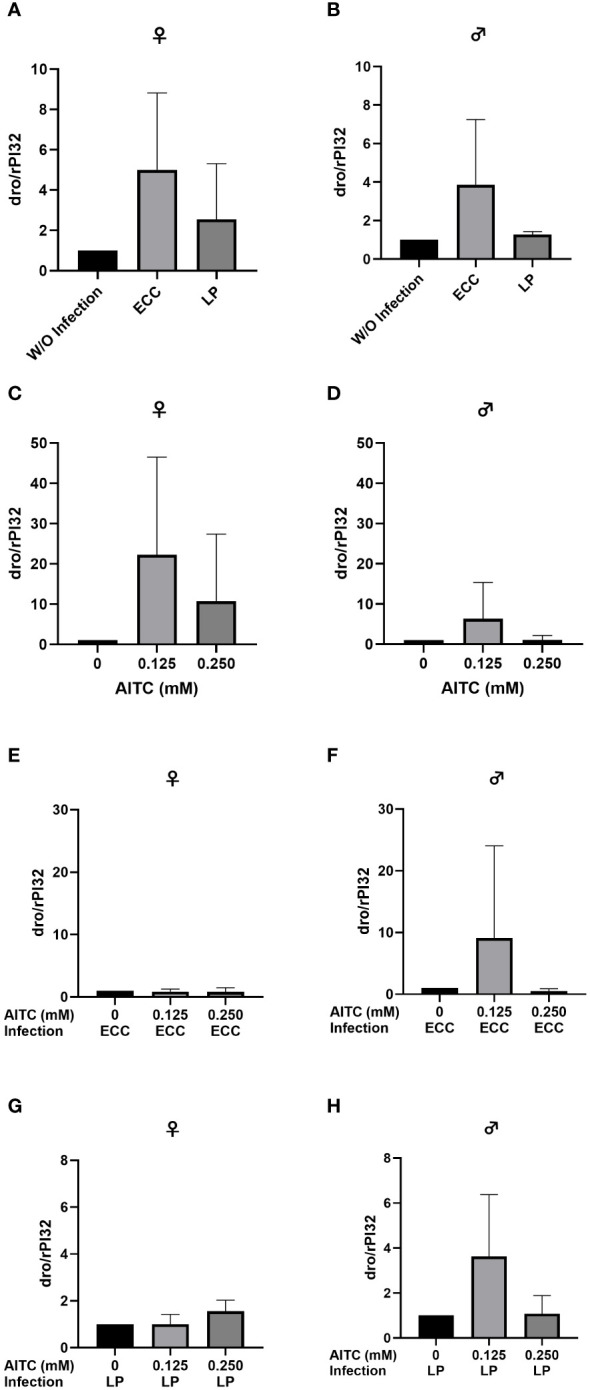
Relative mRNA expression levels of *dro* in female **(A, C, E, G)** and male **(B, D, F, H)** *D. melanogaster* initially kept on either a control or an AITC-supplemented diet (0.125 mM or 0.250 mM) for 10 days and subsequently exposed to a 100-mM sucrose solution with or without *L. pseudomesenteroides* (LP) or *P. carotovorum* subsp*. carotovorum* (ECC) for 18 h. mRNA levels were determined in fly samples from three independent experiments with three replicates containing 10 flies each. Bars show the mean ± SD. Significant differences between groups were tested by applying one-way ANOVA followed by Dunnett’s multiple-comparison test. Significance was accepted at p < 0.05.

**Figure 11:**
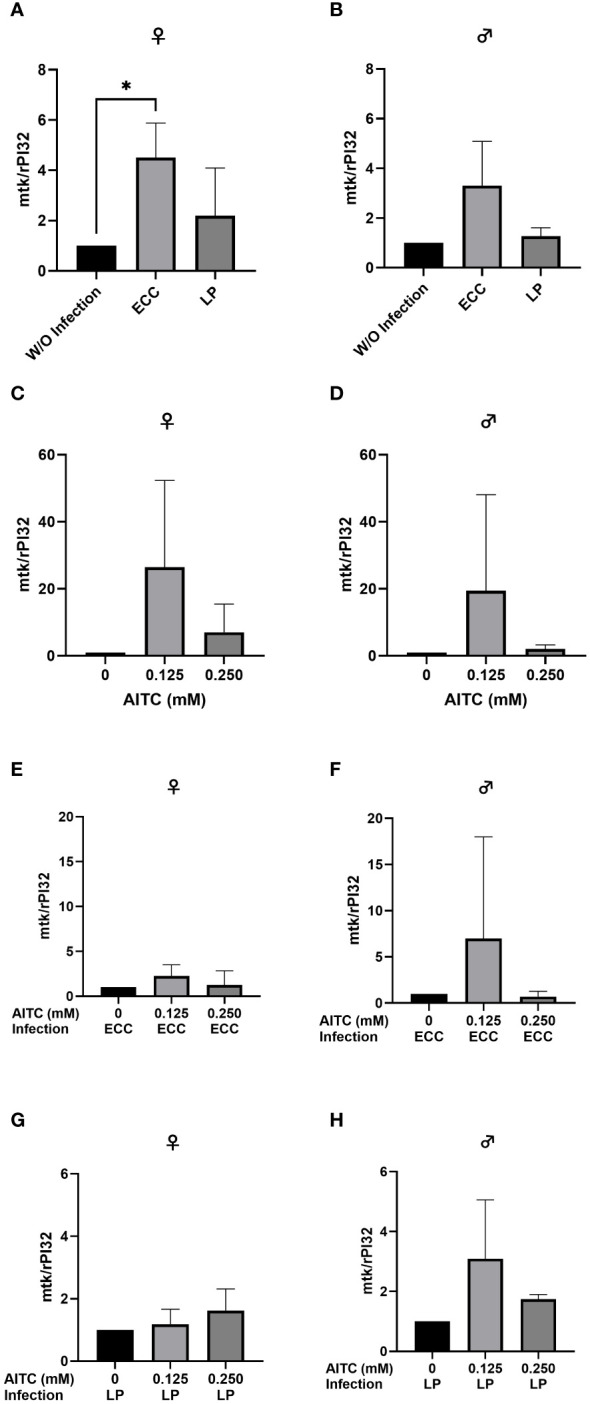
Relative mRNA expression levels of *mtk* in female **(A, C, E, G)** and male **(B, D, F, H)** *D. melanogaster* initially kept on either a control or an AITC-supplemented diet (0.125 mM or 0.250 mM) for 10 days and subsequently exposed to a 100-mM sucrose solution with or without *L. pseudomesenteroides* (LP) or *P. carotovorum* subsp*. carotovorum* (ECC) for 18 h. mRNA levels were determined in fly samples from three independent experiments with three replicates containing 10 flies each. Bars show the mean ± SD. Significant differences between groups were tested by applying one-way ANOVA followed by Dunnett’s multiple comparison test. Significance was accepted at p < 0.05. *p < 0.05.

In order to test for potential changes in the production of AMPs, gene expression levels of *dro* and *mtk* were examined. Neither in flies infected with ECC or LP for 18 h (**Figures 8A, B** and **9A, B**) nor in flies kept on an AITC-supplemented diet for 10 days (**Figures 8C, D** and **9C, D**) effects on either *dro* or *mtk* mRNA expression levels were observed. Flies initially kept on a control diet or an AITC-supplemented diet for 10 days with or without a subsequent bacterial infection did not show any significant changes in *dro* or *mtk* mRNA levels. This applied to infections with both bacterial strains and to both sexes (**Figures 10A–H** and **11B–H**). Only ECC-infected female flies, pre-exposed to an AITC-supplemented diet for 10 days, showed a significant 4.5-fold increase of *mtk* expression levels compared with the uninfected controls (**Figure 11A**) whereas no significant effect was detected for *dro* expression levels (**Figure 10A**).

## 4 Discussion

The rise of bacterial resistance to antibiotics shows a need to identify alternative therapies. Antimicrobial peptides (AMPs) are emerging as promising candidates due to their potent antimicrobial properties (23, 30). Modulating the production of endogenous AMPs, as being already shown for the isothiocyanate sulforaphane *in vitro*, may offer a possibility for preventing bacterial infections (16).

We therefore asked whether AITC may be a potential candidate compound in the treatment of bacterial infections and whether it affects the endogenous production of AMPs. To test health-promoting effects, we initially analyzed the survival of fruit flies exposed to AITC and/or pathogenic bacteria. In male flies, we did not detect any effect of both applied AITC concentrations without the presence of pathogenic bacteria on survival time, neither in a short-term nor in a long-term treatment (**Figures 4C**, **6C**). Intriguingly, we did not detect any effect of AITC on the food intake in female flies but in male flies where 0.250 mM AITC in the diet resulted in a significantly lower food intake (**Figure 3B**). This may explain the unaltered survival curves following AITC exposure in males compared with females where a long-term treatment with AITC resulted in a significant and dose-dependent decrease of the survival rates (**Figure 4B**). Due to the lower food intake of male flies, they potentially also consume less AITC, which could explain the difference in mortality compared with female flies. In addition, in male flies, AITC may have induced a kind of caloric restriction, which is well documented to increase lifespan in *D. melanogaster* (31). Interestingly, Merinas-Amo and colleagues (32) tested different cultivars of *Brassica rapa* with a high difference in their individual GLS and consequently isothiocyanate content on effects on the lifespan of *D. melanogaster*. The authors observed a significant dose-dependent increase of the flies’ lifespan, which was in contrast to the results of the present study where a significant decrease in the lifespan of female flies (**Figure 4B**) and no effect on male flies following exposure to AITC (**Figure 4C**) was detected. These differences may have occurred from the fact that our flies received the isolated isothiocyanate (and not the corresponding precursor sinigrin) via the diet and our results were generated sex-specifically.

One explanation for the detected higher mortality in female flies may have resulted from the applied AITC concentrations that were possibly too high. Insecticidal effects of AITC have been described for other insects but not for *D. melanogaster* (33–36). However, it should be considered that the increased mortality in most studies was caused by AITC applied as a volatile substance. Numerous positive effects were observed in other organisms after an oral ingestion of AITC or AITC-containing extracts possibly due to the intestinal metabolization of AITC (37–42).

However, Mazari et al., 2014 (43) did not detect any weakness or impaired phenotype in wild-type fruit flies exposed to 1 mM AITC in their diet. However, male flies being transgenic for GSTE7, an enzyme important for detoxifying xenobiotics, had a significantly higher mortality when exposed to phenethyl isothiocyanate for 1 week compared with female GSTE7 transgenic flies (43). This may explain the detected differences in the lifespan between male and female flies according to AITC treatment in the present study. Potentially, our male flies had higher GSTE7 levels compared with female flies resulting in an improved detoxification of AITC, which caused the better survival of male compared with female flies.

Although this does not support a potential toxic effect of AITC in our flies, it may be the reason for unchanged *mtk* and *dro* mRNA levels following the exposure to AITC. To examine if AITC exhibited health benefits and affected the mRNA expression of AMPs in pathogen-infected *D. melanogaster*, we orally applied the fly pathogenic bacteria ECC and LP to both female and male *D. melanogaster*. In order to study host–pathogen interactions including the production of AMPs, ECC-infected *D. melanogaster* have already been used (44, 45). Piegholdt et al. (45) showed that ECC induced the mRNA expression levels of the AMPs *mtk*, *dro*, *attB* (attacin B), *attC* (attacin C), and *dpt* (diptericin). In addition, ECC exposure reduced the survival of our *D. melanogaster* under nutrient deficiency (**Supplementary Figure S1**), as shown for other bacteria (46, 47). For LP-mediated infections, to the best of our knowledge, there is currently only information for bacteriocins produced by LP but no information on a potential effect on AMP expression levels available. However, LP infection also reduced the survival of fruit flies under nutrient deficiency (**Supplementary Figure S1**), which supports the findings of Hiebert et al. (47). In case of an oral 18-h-infection of 3-day-old female *D. melanogaster* under sufficient nutrient supply, we observed a significant reduction in survival time following ECC treatment (**Figure 4D**). In contrast, an infection of 13-day-old female flies had no effect on survival (**Figure 6D**). In male flies, an infection with LP at the age of 3 days and an infection with ECC at the age of 13 days even resulted in a significant increase in survival time (**Figure 4E** and **Figure 6E**). This may be due to a sex-specific response to bacterial infections. It was demonstrated that in general males and females in various species differ in their immunological responses. When challenged with antigens, females generally have a more protective humoral and cell-mediated immune response, whereas for males, more intense inflammatory immune responses were described (48, 49). In *D. melanogaster*, female flies were described to be more likely than male flies to die from infections with several strains of *Beauveria bassiana*, which was postulated to be substantiated from sex-specific differences in the toll and the Imd pathway (50). These pathways are activated by bacteria and fungi consequently inducing the production of AMPs in *D. melanogaster* (27), which only owns an innate immune system (21). The negative effect of AITC on the survival time of female *D. melanogaster* appeared to reinforce the influence of ECC, as an AITC dose-dependent reduction in survival time was observed in both, in an infection followed by long-term treatment with AITC (**Figure 5D**) and in a 10-days treatment with AITC followed by a subsequent infection (**Figure 7D**).

When looking at the expression levels of AMPs, no significant differences were detected for *dro* and *mtk* after an 18-h-infection with ECC and LP of 3-day-old female and male flies (**Figure 8A** and **Figure 9A**). However, it has to be considered that larger variations between the experimental replicates may have masked a potential increase in AMP expression levels. Since we analyzed the AMP expression in the whole organism, a detection of AMP levels in individual organs might have reduced the observed variations in our data. In contrast, the infection of 13-day-old females with ECC led to a significant higher expression level of *mtk* (**Figure 11A**). Rera et al. (51) showed that the expression of AMPs was tightly related to a dysfunction of the intestinal barrier, which increased in the course of life and therefore caused higher AMP levels in older flies (52–54). These adverse effects on the intestinal barrier may be further increased by pathogenic infections. With regard to the AMP expression levels in our flies being exposed to AITC, no effect of both AITC concentrations was observed (**Figures 8**-**11**). This was also true for additional bacterial infections, where AITC was applied after oral bacterial infections (**Figures 10**, **11**). Perhaps the larger variations between the experimental replicates, which could be due to differences between the individual flies, masked a potential statistically significant difference between the AMP expression levels.

In female fruit flies, the response to infections also seems to depend on whether they have already mated. If there was only a limited amount of energy available, the fly had to decide where to invest the energy—either in the process of reproduction or in the immune defense. Experiments conducted by Gordon and colleagues (55) support this assumption since infected mated female flies had a lower rate of survival, a higher bacterial load, and lower AMP levels in comparison with unmated females. As mated female flies were used in our experiments this could—at least partly—explain why we did not detect many differences in the expression levels of *dro* and *mtk* in the infected flies.

Overall, the results of the present study indicate that AITC treatments caused a significant decrease in survival among females but not males. Furthermore, AITC reduced the survival of females even in the presence of ECC, but not in the presence of LP, whereas no effect of AITC on survival rates were observed in infected male flies. Similarly, AITC did not significantly affect AMP levels in either infected male or infected female flies. The observed differences in survival between female and male flies may depend on sex-specific variations in immune responses as evidenced by the differing outcomes following bacterial infection in males. The high variations in mRNA expression levels of AMPs due to differences between experimental replicates may have obscured significant differences. Therefore, further experiments are needed to investigate the impact of AITC on pathogenic bacterial infections in fruit flies in more depth and with a specific focus on inflammatory signaling pathways.

## Data availability statement

The original contributions presented in the study are included in the article/**Supplementary Material**. Further inquiries can be directed to the corresponding author.

## Ethics statement

Ethical approval was not required for the study involving animals in accordance with the local legislation and institutional requirements because study included the use of lower invertebrate animals (*Drosophila melanogaster*).

## Author contributions

CZ: Conceptualization, Data curation, Formal analysis, Investigation, Methodology, Writing – original draft, Writing – review & editing. SD: Investigation, Methodology, Writing – original draft, Writing – review & editing. AW: Conceptualization, Project administration, Supervision, Writing – original draft, Writing – review & editing.
