# Supplementary material for: Effect of allyl-isothiocyanate on survival and antimicrobial peptide expression following oral bacterial infections in Drosophila melanogaster
Source: Front Immunol. 2024 May 13;15:1404086. doi: 10.3389/fimmu.2024.1404086 (PMC11128604; doi:10.3389/fimmu.2024.1404086)
Supplement: Supplementary file 1 [file DataSheet_1.docx]

Supplementary Figure

#

**Figure S1:** Survival curves and median survival time of female **(A)** and male **(B)** *D. melanogaster* exposed to control solution (W/O Infection) or a solution with *L. pseudomesenteroides* (LP) or *P. carotovorum* subsp. *carotovorum* (ECC) until the end of their lifetime. Results show the three independent experiments. Significant differences between treatments were tested by the Kaplan-Meier approach and a log-rank test. Significance was accepted at p<0.05. *p<0.05, ****p<0.0001.
